# IMPROVEMENTS NEEDED FOR JAPANESE ELDER ABUSE PREVENTION LAW: COMPARISONS WITH OTHER ABUSE PREVENTION LAWS

**DOI:** 10.1093/geroni/igz038.1684

**Published:** 2019-11-08

**Authors:** Asako Katsumata, Noriko Tsukada

**Affiliations:** 1 Higashigaokaso rehabilitation institution, Japan; 2 Nihon University, Tokyo, Japan

## Abstract

Japan has four stand alone abuse prevention laws, including child abuse (enforced in 2000), domestic abuse (2001), elder abuse (2006) and abuse for people with disabilities (2011). These laws are distinctive in that they aim to prevent abuse, not merely to address abuse after it occurs. This paper compares components of these four abuse prevention laws, delineating major strengths and weaknesses of the elder abuse prevention law in comparison to the other three. The analysis considers both institutional and domestic settings and suggests possible improvements of elder abuse prevention law that need to be made. Evidence for this analysis is supplied through examination of trends abuse using longitudinal data (2012-2017) collected by Japan’s Ministry of Health, Labour and Welfare. The analysis shows commonalities in trends in the four abuse categories. For example, the number of abuse cases has risen over time despite the dissemination and implementation of abuse prevention training, program implementation, and public outreach. Differences include reporting – police more often report abuse cases of children and people with disabilities, while elder abuse cases are more often reported by professional staff members responsible for dealing with elder abuse cases. Although some amendments have been made to the child abuse and domestic abuse prevention laws, no amendments have been made to the elder abuse prevention law despite the requirement to review its success. Needed revisions include provisions of protection orders and temporary shelters to protect elder victims from abusers as soon as possible.

